# Collecting core data in physician-staffed pre-hospital helicopter emergency medical services using a consensus-based template: international multicentre feasibility study in Finland and Norway

**DOI:** 10.1186/s12913-019-3976-6

**Published:** 2019-03-08

**Authors:** Kristin Tønsager, Marius Rehn, Kjetil G. Ringdal, Hans Morten Lossius, Ilkka Virkkunen, Øyvind Østerås, Jo Røislien, Andreas J. Krüger

**Affiliations:** 1 The Norwegian Air Ambulance Foundation, Oslo, Norway; 2 Department of Anaesthesiology and Intensive Care, Stavanger University Hospital, Stavanger, Norway; 3 Department of Health Studies, University of Stavanger, Stavanger, Norway; 4 Pre-hospital Division, Air Ambulance Department, Oslo University Hospital, Oslo, Norway; 5 Department of Anesthesiology, Vestfold Hospital Trust, Tønsberg, Norway; 6 Division of Emergencies and Critical Care, Department of Anesthesiology, Oslo University Hospital, Oslo, Norway; 7 Norwegian Trauma Registry, Oslo University Hospital, Oslo, Norway; 8 Research and Development Unit, FinnHEMS, Vantaa, Finland; 9 Department of Anaesthesiology and Intensive Care, Haukeland University Hospital, Bergen, Norway; 10 Department of Emergency Medicine and Pre-Hospital Services, St. Olavs Hospital, Trondheim, Norway

**Keywords:** Critical care, Emergency medical services, Pre-hospital emergency care, Feasibility studies, Documentation, Data collection

## Abstract

**Background:**

Comparison of services and identification of factors important for favourable patient outcomes in emergency medical services (EMS) is challenging due to different organization and quality of data. The purpose of the present study was to evaluate the feasibility of physician-staffed EMS (p-EMS) to collect patient and system level data by using a consensus-based template.

**Methods:**

The study was an international multicentre observational study. Data were collected according to a template for uniform reporting of data from p-EMS using two different data collection methods; a standard and a focused data collection method. For the standard data collection, data were extracted retrospectively for one year from all FinnHEMS bases and for the focused data collection, data were collected prospectively for six weeks from four selected Norwegian p-EMS bases. Completeness rates for the two data collection methods were then compared and factors affecting completeness rates and template feasibility were evaluated. Standard Chi-Square, Fisher’s Exact Test and Mann-Whitney U Test were used for group comparison of categorical and continuous data, respectively, and Kolomogorov-Smirnov test for comparison of distributional properties.

**Results:**

All missions with patient encounters were included, leaving 4437 Finnish and 128 Norwegian missions eligible for analysis. Variable completeness rates indicated that physiological variables were least documented. Information on pain and respiratory rate were the most frequently missing variables with a standard data collection method and systolic blood pressure was the most missing variable with a focused data collection method. Completeness rates were similar or higher when patients were considered severely ill or injured but were lower for missions with short patient encounter. When a focused data collection method was used, completeness rates were higher compared to a standard data collection method.

**Conclusions:**

We found that a focused data collection method increased data capture compared to a standard data collection method. The concept of using a template for documentation of p-EMS data is feasible in physician-staffed services in Finland and Norway. The greatest deficiencies in completeness rates were evident for physiological parameters. Short missions were associated with lower completeness rates whereas severe illness or injury did not result in reduced data capture.

**Electronic supplementary material:**

The online version of this article (10.1186/s12913-019-3976-6) contains supplementary material, which is available to authorized users.

## Background

Systems for pre-hospital critical care exist worldwide, but emergency medical service (EMS) systems differ in resources, organizational and operational models; from simple systems providing basic life support to sophisticated systems providing critical care [1–10].

Treatment and diagnostic options in pre-hospital care are increasing and several in-hospital techniques are currently being applied in the pre-hospital setting [10–14]. To enable more point-of-care diagnostics and increase in advanced interventions, some EMS systems, especially in Europe and Australasia, have introduced helicopters and rapid-response cars staffed with specially trained physicians [10, 15]. The effect of physician-staffed EMS (p-EMS) is debated and studies report contradicting results [16–28]. A substantial challenge to assess quality of health care is lack of uniform documentation, this is also pertinent to p-EMS [29, 30].

The concept of consensus-developed condition-specific datasets has proven useful for research and quality assessment in several areas of critical care [30–33]. To evaluate the effect and efficiency of p-EMS, a template for uniform reporting of data from p-EMS was published [34]. However, to implement a template for documentation, feasibility of the template to collect the requested data in the context intended should be demonstrated [29, 35–39].

In Scandinavia, p-EMS is well established, and services are relatively similar, thus joint research efforts may be valuable [5, 34]. Finland is currently the only country where the template for documenting and reporting from p-EMS is implemented, thus the only country able to provide routinely collected template data. To evaluate template feasibility, we wanted to compare two different data collection methods in two similar systems. P-EMS in Finland and Norway employ the same operational and medical concept and differences between services are mainly seen in time variables, patient volume and service area [5]. We considered comparison of Finland and Norway to be feasible; thus, we decided to include these two countries for the present study.

The aim of the present study was to evaluate the feasibility of pre-hospital physicians to collect patient and system level data by using the template for uniform reporting of data from p-EMS [34], comparing data collection from a standard to a focused data collection method.

## Methods

### Study design

The study was an international multicentre feasibility study including two physician-staffed pre-hospital services. As the aim of the study was to examine the feasibility of collecting template data in a standard operational pre-hospital context, we designed a two-method collection protocol. We hypothesized that by using a dedicated and motivated group of physicians (focused data collection method), we would achieve a robust indication of whether the template data were possible to collect in general. By comparing data collected with the focused collection method to routinely collected data (standard data collection method) we could assess whether both methods were feasible, or if data collection was feasible for specially dedicated physicians only.

For the standard data collection method, data from the five p-EMS bases administered by FinnHEMS (the national operator of p-EMS in Finland), covering a total population of 3.7 million inhabitants, were extracted from their database for a period of 12 months (March 2013 through February 2014). The physicians were not informed that data were extracted, thus completeness rates represents routinely collected data for FinnHEMS.

For the focused data collection method, template data were collected prospectively for six weeks in Norway (January through March 2014) by 16 physicians from four p-EMS bases, covering a total population of 1.75 million inhabitants. Each participating physician was asked to collect template data as complete as possible on a predefined form and all physicians were informed that this was a study of completeness rates. Emphasis was on keeping the data collection period short to avoid study-fatigue. Data were placed in standardized categories and data sets from Finland and Norway were then merged.

Feasibility of the two data collection methods were assessed by comparing completeness rates on several variables. Variables that proved difficult to collect were identified and reasons for different completeness rates were sought by comparing completeness rates for different patient groups and operational settings. Data were stratified according to medical problem, p-EMS escort to hospital, severity of the patient’s condition, patient age, time from p-EMS arrival on scene to delivery at hospital and mode of transportation.

Strengthening the Reporting of Observational Studies in Epidemiology (STROBE) [40] and Standards for Quality Improvement Reporting Excellence (SQUIRE) [41] guidelines were consulted when drafting the manuscript.

### Data variables

The template for data collection consists of five main sections [34]. The first section, “Fixed system variables” contain data about service area, organization and activation criteria and is identical to all missions for each base, hence this section was not included in the study. The second section, “Event operational descriptors”, contain time data, data on dispatch and type of transportation. The third section, “Patient descriptors”, contain patient data, data on patient physiology and medical problem. The fourth section, “Process mapping data”, contain data on medication and procedures performed during the mission and the fifth section, “Outcome measures”, contain data on mission outcome. A full description of all variables is provided in Additional file 1. Physicians in Norway were instructed to register event operational and patient descriptors, process mapping and outcome measures. In total 33 variables were registered. Information on gender was omitted to de-identify patients. Further, the outcome measure “Physiological improvement” was also omitted, as this is a proposed quality indicator yet to be validated. The corresponding variables were extracted from the FinnHEMS database. For the standard data collection method, all process mapping data and data on unit arrival at scene, type and result of dispatch, comorbidity and medical problem were mandatory to register to complete patient records. For the focused data collection method, no variable was mandatory.

### Statistical analysis

The two data collection methods were compared by comparing completeness rates on several variables. Variables that proved difficult to collect were identified and reasons for different completeness rates were sought by comparing completeness rates for different patient groups and operational settings. Data were stratified according to medical problem, p-EMS escort to hospital, severity of the patient’s condition, patient age, time from p-EMS arrival on scene to delivery at hospital and mode of transportation. Categorical data are presented as counts (n) and proportions (%) while continuous data are presented as median and interquartile range (IQR). Standard Chi-Square, Fisher’s Exact Test and Mann-Whitney U Test were used for group comparison of categorical and continuous data. Kolomogorov-Smirnov test [42] was used for comparison of distributional properties. Data were analyzed using IBM SPSS statistics version 22 and R 3.1.0.

## Results

### Study material

FinnHEMS submitted data from 12,486 missions. Of these, 8049 (64%) missions were excluded due to no patient encounter (supervision or advice only or due to a concurrent mission, weather or technical conditions), leaving 4437 (36%) missions eligible for further analyses. Norwegian p-EMS submitted data from 177 missions. Of these, 49 (28%) missions were excluded because of no patient encounter (due to weather or technical conditions), leaving 128 (72%) missions eligible for further analyses. The physicians in Norway registered on average 8 forms each, which is 1–2 forms per shift.

### Patient and mission characteristics

Patient and mission characteristics are summarized in Table 1. In both countries the majority of dispatches were for medical missions. Finland had more trauma dispatches than Norway but fewer inter-hospital transfers. In both countries, trauma was the single most common medical problem, followed by cardiac arrest in Finland and chest pain in Norway. In Finland, p-EMS physicians were transported to the scene by helicopter, but most patients were transported to the hospital by ground ambulances accompanied by the p-EMS physician. Most Norwegian patients were transported to the hospital by helicopter. Finland had significantly longer median on-scene time and median time from origin call to patient arriving hospital compared with Norway, but there was no difference in transport time or time from when call was received at the emergency medical communication centres to p-EMS arrival at scene. Significantly more advanced procedures were performed in Finland compared to Norway, but there was no difference in the number of patients receiving medication.

**Table 1:** Patient and mission characteristics. Table depicts number of missions with registered variables and percent of registered variables

	Finland (*n* = 4437)	Norway (*n* = 128)	*p*-value^1^
Type of dispatch			
1 = Medical mission	2509 (57%)	77 (60%)	0.417
2 = Trauma mission	1687 (38%)	33 (26%)	0.005
3 = Inter hospital transfer	80 (2%)	17 (13%)	< 0.001
4 = Search and rescue mission	0	1 (1%)	< 0.001
5 = Consultation	161 (4%)	0	0.028
6 = Other	0	0	
Missing	0	0	
Type of transportation			
1 = Ground ambulance	3599 (81%)	35 (27%)	< 0.001
2 = Helicopter ambulance	95 (2%)	80 (63%)	< 0.001
3 = Fixed-wing	0	0	
4 = Other	0	0	
5 = No transportation	723 (16%)	4 (3%)	< 0.001
Missing	20 (0.5%)	9 (7%)	
Age			
Median (IQR)	58 (34–73)	54 (0–89)	0.002
Co-morbidity			
1 = No (pre-event ASA-PS = 1)	1213 (27%)	51 (40%)	0.002
2 = Yes (pre-event ASA-PS = 2–6)	2560 (58%)	73 (57%)	0.881
3 = Unknown	664 (15%)	4 (3%)	< 0.001
Medical problem			
1 = Cardiac arrest	889 (20%)	17 (13%)	0.059
2 = Trauma	1313 (30%)	35 (27%)	0.582
3 = Breathing difficulties	259 (6%)	8 (6%)	0.844
4 = Chest pain	128 (3%)	20 (16%)	< 0.001
5 = Stroke	256 (6%)	12 (9%)	0.087
6 = Acute neurology excluding stroke	606 (14%)	15 (12%)	0.528
7 = Psychiatry including intoxications	413 (9%)	2 (2%)	0.003
8 = Obstetrics and childbirth	74 (2%)	2 (2%)	0.927
9 = Infection	45 (1%)	6 (5%)	0.001
10 = Other	446 (10%)	11 (9%)	0.588
On scene time (min)			
Median (IQR)	22 (13–33)	12 (6–20)	< 0.001
Time from origin call to arrival hospital (pre-hospital time interval) (min)			
Median (IQR)	83 (62–109)	72 (49–98)	0.001
Transport time (min)			
Median (IQR)	24 (14–39)	23 (15–33)	0.585
Time call received at emergency medical communication centre – arrival at scene (min)			
Median (IQR)	23 (16–34)	26 (14–44)	0.199
Patients registered with advanced procedures	3064 (69%)	23 (18%)	< 0.001
Patients given medication	2470 (56%)	74 (58%)	0.630
Patients given medication and registered with advanced procedures	2101 (47%)	16 (13%)	< 0.001
Patients given either medication, advanced procedures or both	3433 (77%)	81 (63%)	< 0.001

1: Chi-Square for categorical data and Mann-Whitney U Test for continuous data

*ASA-PS*: American Society of Anaestehesiologists Physical Status classification

*IQR*: Inter Quartile Range

### Completeness of patient-level core data

With the standard data collection method, all 13 mandatory variables were 100% complete (Table 2) while further four of the variables were > 80% complete. Ten variables had < 50% completeness. Six out of ten physiological variables (first and last value of heart rate, systolic blood pressure, heart rhythm, oxygen saturation and respiratory rate) had < 50% completeness. With the focused data collection method, seven variables were 100% complete, and overall 29 variables were > 80% complete. Two variables were < 50% complete. Except from the two variables reporting first and last systolic blood pressure, all physiological variables were > 80% complete.

**Table 2:** Completeness rates for reporting in p-EMS. Table depicts number of missions with registered variables and percent of registered variables for standard and focused data collection method

Data point name	Standard (*n* = 4437) (%)	Focused (*n* = 128) (%)	*p*-value^1^
*Event operational descriptors*			
Call received at emergency medical communication centre	3085 (69.5)	128 (100)	< 0.001
Unit arrival at scene	4437 (100)	128 (100)	NA
Patient leaving scene	2493 (56.2)	117 (91)	< 0.001
Patient arriving hospital	1804 (40.7)	110 (86)	< 0.001
Type of dispatch	4437 (100)	128 (100)	NA
Type of transportation	4417 (99.5)	128 (100)	1.0
Result of dispatch	4437 (100)	128 (100)	NA
*Patient descriptors*			
Age	4434 (99.9)	127 (99)	0.108
Comorbidity	4437 (100)	128 (100)	NA
Medical problem	4437 (100)	128 (100)	NA
Glasgow coma score first	3712 (83.7)	125 (98)	< 0.001
Glasgow coma score last	1843 (41.5)	124 (97)	< 0.001
Heart rate first	2893 (65.2)	119 (93)	< 0.001
Heart rate last	1591 (35.9)	109 (85)	< 0.001
Systolic blood pressure first	2827 (63.7)	99 (77)	0.001
Systolic blood pressure last	1561 (35.2)	90 (70)	< 0.001
Rhythm first	3363 (75.8)	118 (92)	< 0.001
Rhythm last	1533 (34.6)	117 (91)	< 0.001
SpO2 first	2761 (62.2)	110 (86)	< 0.001
SpO2 last	1600 (36.1)	106 (83)	< 0.001
Pain first	863 (19.5)	123 (96)	< 0.001
Pain last	447 (10.1)	122 (95)	< 0.001
Respiratory rate first	2040 (46)	112 (88)	< 0.001
Respiratory rate last	1043 (23.5)	105 (82)	< 0.001
*Process mapping*			
Diagnostic procedures	4437 (100)	53 (41)	< 0.001
Drugs to facilitate airway procedure	4437 (100)	122 (95)	< 0.001
Device for successful airway management	4437 (100)	121 (95)	< 0.001
Breathing – procedures used	4437 (100)	46 (36)	< 0.001
Circulation – procedures used	4437 (100)	126 (98)	0.001
Disability – procedures used	4437 (100)	111 (87)	< 0.001
Medication – drugs administered	4437 (100)	127 (99)	0.028
Type of medication]	4437 (100)	123 (96)	< 0.001
*Outcome Measures and Quality Indicators*			
Mission Outcome	4419 (99.6)	127 (99)	0.418

1: Fisher’s exact test

NA: not possible to calculate as two cells have a frequency of zero

### Completeness rate and patient characteristics

**Fig. 1:**
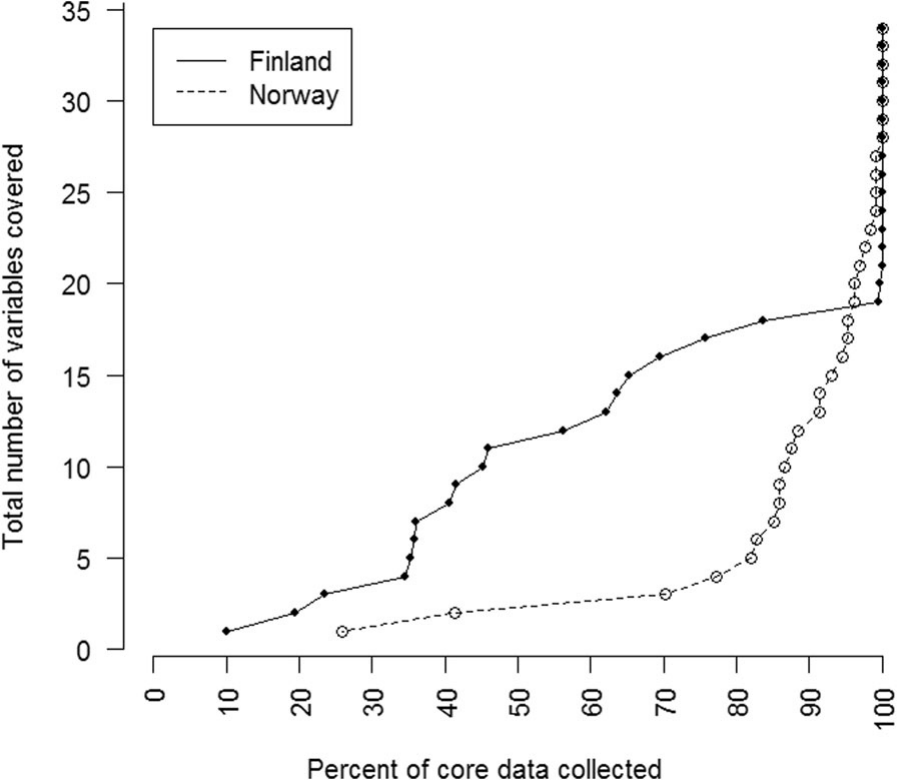
Completeness rates for standard and focused data collection method. Figure depicting completeness rates for all variables with a standard and a focused data collection method. Each dot represents one variable, and the corresponding percent of core data collected for that variable. For perfect collection, the figure would be a vertical line of dots a 100%

**Fig. 2:**
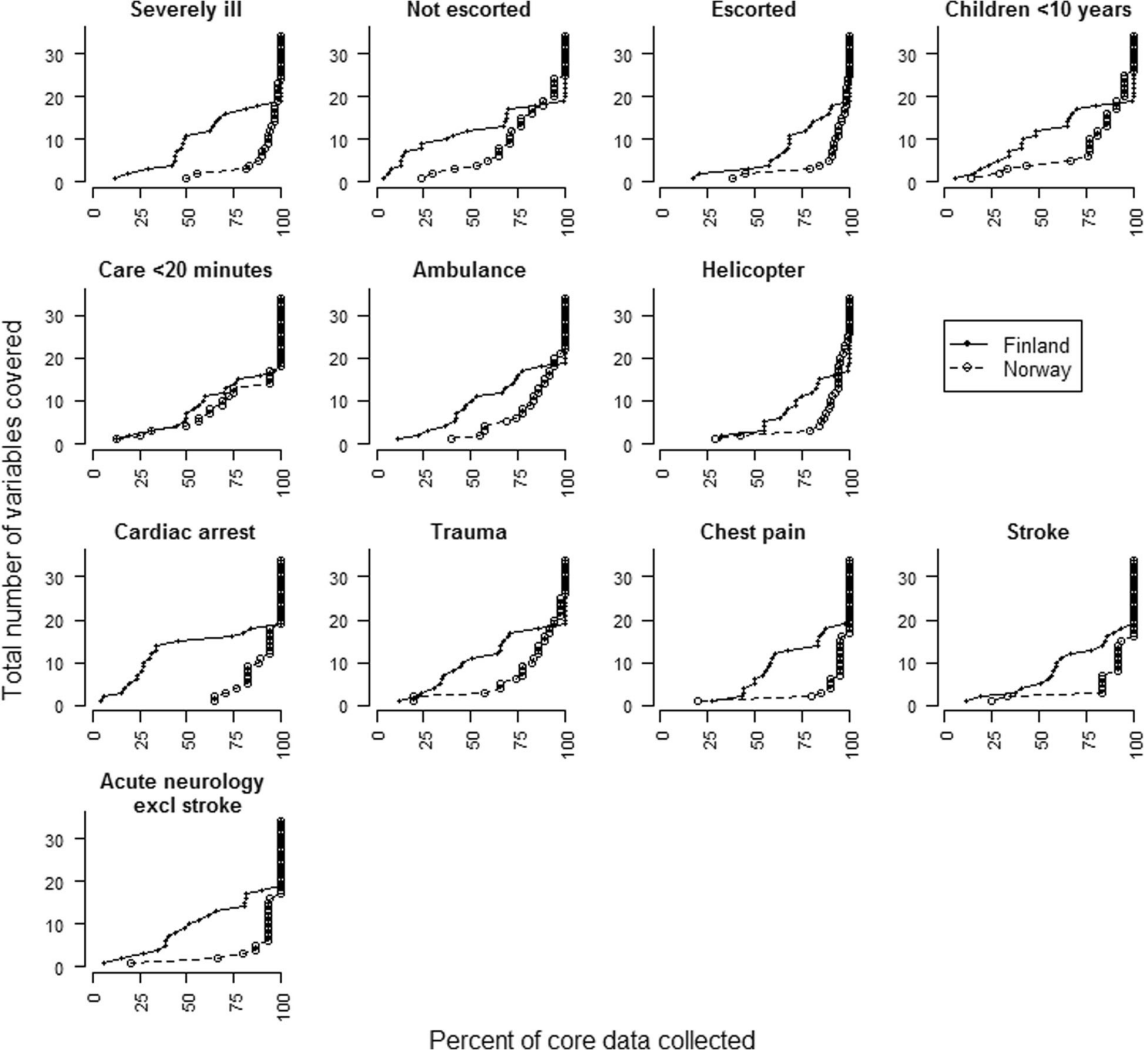
Completeness rate variations for different subgroups. Figure depicting completeness rates for different patient groups, operational characteristics and medical conditions for standard and focused data collection methods. Each dot represents one variable, and the corresponding percent of core data collected for that variable. For perfect collection, the figure would be a vertical line of dots a 100%

Completeness rates were affected by clinical problems encountered and mission characteristics. Significantly more variables were collected with a focused data collection method than with a standard data collection method, both for different medical conditions, when patients were severely ill or injured and when patient care was less than 20 min. An additional file (Additional file 2) depicts our definition of a severely ill or injured patient. More variables were collected with focused data collection, regardless of transport mode. Completeness rate variations among different clinical problems are depicted in Figs. 1 and 2 and Table 3.

**Table 3:** Completeness rates per patient group, operational characteristics and medical conditions. Table depicts percent of data documented with standard and focused data collection method

	Standard (%)	Focused (%)	*p*-value
Cardiac arrest	66	92	< 0.001
Trauma	73	85	< 0.001
Chest pain	80	94	< 0.001
Stroke	80	91	< 0.005
Acute neurology	75	93	< 0.001
Severely ill	75	93	< 0.001
Care ≤20 min	82	88	< 0.001
Transport: Ambulance	75	87	< 0.001
Transport: Helicopter	91	85	0.002

When comparing different patient groups for each data collection method, we found that for both methods, significantly less variables were collected when patient care was less than 20 min than when patient care was more than 20 min. Further, to be escorted by a physician to hospital resulted in more reported variables than when patients were treated by physicians on-scene and transported without physician. For children under 10 years of age, less variables were collected than when patients were older. Transport by helicopter resulted in significantly higher completeness rates with a standard data collection method, but there was no significant difference regarding transport mode with a focused data collection method. With standard data collection, significantly more variables were collected when patients were severely ill or injured compared to not severely ill or injured patients. There was no significant difference among these patient groups with a focused data collection. Differences in completeness rates among different patient groups with the two data collection methods are summarized in Table 4.

**Table 4:** Comparison of completeness rates for different patient groups. Table compare different patient groups with standard and focused data collection method

	Standard (%)	*p*-value^1^	Focused (%**)**	*p*-value^1^
Severely ill or injured vs. not severely ill or injured	75 vs. 66	< 0.001	93 vs. 87	0.094
Care ≤20 min. vs. care > 20 min.	79 vs. 84	< 0.001	82 vs. 93	0.009
Escorted by physician vs. not escorted by physician	83 vs. 68	< 0.001	93 vs. 80	0.017
Transport ambulance vs. transport helicopter	75 vs. 85	< 0.001	87 vs. 91	0.394
Patients ≤10 years old vs. > 10 years old	70 vs. 73	0.030	82 vs. 91	0.008

1: Kolomogorov-Smirnov-test

## Discussion

When efforts are optimized, p-EMS can achieve high completeness rates in collecting prospective data using a template. Motivation and focus on documentation, rather than operational context, seems to affect data completeness rates most.

Lack of documentation is often highlighted as a limitation for research in emergency medicine, especially for retrospective registry studies [5, 35, 43–45]. Putting attention to increase the quality of routinely collected data may enable such data to be an important and effective source to monitor and compare services. As such, strategies to increase data capture should be sought [30, 43, 45–48]. Training programs may increase data capture, most likely by increasing attention to documentation [43, 48]. In our study the effect of motivation was evident, where significantly more data were registered with a focused data collection method than with a standard data collection method. Feedback on how high-quality research or quality assurance will benefit from complete data registration can make physicians more aware of the importance of data registration, thereby increasing data capture.

Echoing our results, several studies have found physiological variables to be the least documented variables [44, 45, 49]. Laudermilch et al. [44] found that 28% of patient records had missing physiological data and Bergrath et al. [45] reports vital parameters necessary to document Mainz Emergency Evaluation Score (MEES) to be present at two time points in only 31.08% of patients. Gravel et al. reports from the paediatric population that high rates of vital signs data are missing [50]. With a standard and a focused data collection method, 48 and 85% of physiological variables were registered, respectively, indicating that high completeness rates are achievable. However, physiological data were not complete, even with a focused data collection method. Good clinical assessment depends on correct evaluation of vital signs; thus, documentation of physiological variables is important [48, 50] and strategies for improvement of reporting should be sought.

Physiological data change according to patient state and repeated registrations of the same variable capture trends and reveal changes in patient condition and the effect of treatment [30–32, 46, 47, 51]. The p-EMS template requests documentation of physiological variables at two time points. For all repeated parameters we found the first value to be more complete than the last value, thereby complicating intervention comparison and comparison of changes in patient state. This is comparable with the findings of Bergrath et al. [45]. Medical directors should emphasize the statutory requirement for temporal documentation of physiological parameters and that this also pertains to p-EMS [52].

Ideally data capture in pre-hospital critical care should be simple, accurate and fast. For both cohorts, clinical data are registered on paper during the mission and are later digitally registered. This process is time-consuming, inexpedient and carries a risk for recall-bias and documentation fatigue. Automated data capture from monitors may increase completeness rates and is widely used in anaesthetic services documenting every change in the patient state [53]. Implementation of these readily available concepts to the pre-hospital environment is increasing [54]. Although there are still challenges, automated data capture may reduce administrative workload, improve patient focus and transferal of patient documentation to the next level of care [54–58].

Laudermilch et al. [44] suggests that datasets are less complete for more severely injured patients and that increased workload reduce data capture. This is in contrast to our findings, where data capture was increased or remained equal for patients with a critical condition (Table 3). Corresponding with our findings, Bergrath et al. report calculability of MEES to improve with increasing medical severity [45]. Patients with minor complaints might be considered to require less attention and thereby an increased amount of missing data occurs [49, 59, 60]. However, with a focused data collection method, we found no differences in data completeness for less critical patients.

Time available for data capture may affect completeness rates. We found missions with less than 20 min of patient encounter were associated with lower completeness rates than missions lasting more than 20 min. This may reflect increased workload. For children below 10 years of age, we found lower completeness rates of vital parameters than for patients above 10 years of age. For less severely ill or injured children, measuring blood pressure can be uncomfortable and doctors may be reluctant to perform the measurements, resulting in lower completeness rates.

Categorization of data may increase data capture compared to registering exact values [59]. In Finland, where data were collected by a standard method, the template has been modified and pain was reported using a scale from 1 to 10 instead of using the original three-parted scale described in the template; no pain, moderate pain and severe pain. Jennings et al. [61] recommends the verbal numerical rating scale to measure pain in the pre-hospital setting, corresponding with the FinnHEMS template modification. In Norway, where a focused data collection method was used, pain was reported according to the original template. With the standard data collection method, we found that completeness rates for data on pain were low while with the focused data collection method, data on pain were almost complete, supporting a reduced number of categories to increase data capture. However, fewer categories reduce precision, leading to imprecise estimates, and must be weighed against the need for accuracy.

Outcome comparison often adjust for on-scene time, making low documentation completeness or imprecise registrations of this variable a limitation for research [62–64]. Eckstein et al. [65] found on-scene time being documented in 70% in a cohort of major trauma patients. In our study, on-scene time was documented in 56% an 91% of the cases with a standard and focused data collection method respectively, indicating that high completeness rates are achievable when attention is directed towards documentation. In Norway, the emergency medical communication centres automatically documented the origin time data whereas the response units registered other time variables on paper or non-portable devices [59]. Due to weather and operational conditions, paper registration was often not feasible, and variables were often registered in retrospect, increasing the risk for imprecise registrations. Portable devices available for registrations on site could further increase completeness rates and accuracy of data.

In our data Finland report significantly longer median on-scene times than in Norway (22 versus 12 min), still on-scene times are considerable shorter than reported from German (32 min) and Dutch (27.2 min) services [66]. For trauma patients, the concept of aiming for a pre-hospital time period less than one hour (“The golden hour”) and of keeping on-scene times to not more than 10 min, have been directional for organization of pre-hospital care [67]. In recent years these concepts have been challenged [67–69]. Harmsen et al. conclude that emphasis should be on making sure the patient receives proper pre-hospital care rather than on getting the patient to hospital as fast as possible [70]. In our data, physicians in Finland are providing significantly more advanced procedures than in Norway. This may explain the longer on-scene times in Finland. We do not know, for our system, which advanced procedures should be performed by pre-hospital physicians to improve patient care. However, we believe that uniform documentation may enable us, in the future, to identify procedures beneficial in p-EMS.

All process mapping data (procedures performed, and medication administered) are mandatory in Finland, possibly explaining the 100% completeness rates. In Norway, where no data points are mandatory, completeness vary between 26 and 99% for process data.

Two variables showed particularly low completeness rates in Norway: “Diagnostic procedures” and “Breathing – procedures used”. For these two variables there is no option for choosing “none” or “not relevant”, and when no procedure is performed these data fields will appear as missing. We suggest this to be revised in the template.

Comparing data from two countries had some practical challenges. Although both data collection methods collected data according to the same template, the data were registered in different data formats and in different language. To be able to compare the datasets a work-intensive data management job was needed to standardize categories. Thus, to allow rapid and accurate comparison we recommend data to be registered in the same data format. This is achievable and one might suggest a digital template with predefined names and categories to be implemented. This means that data, when transformed into statistical analysis software, must have the same properties, names and limitations to be able to be easily merged into the same database and analyzed. Adaptions where additional variables are included for local purposes can easily be managed within such a digital template without hampering template comparisons. We believe that simplifying the comparison processes by standardizing data entry will generate more multi-centre research.

### Limitations

The present study has several limitations. We did not include a formal questionnaire to investigate reasons for missing data, although most physicians in Norway provided informal information regarding this. A questionnaire could have been useful to discover reasons for registration failure of importance to aid revision of the template. In Finland, the physicians were not informed about the study in advance, so the database reflects normal documentation rates in FinnHEMS bases. In Norway, the Hawthorne effect is an obvious and wanted effect, whereas the risk for this in Finland is lower.

The data are from 2013/2014 and this may be considered old. However, documentation method or organisation of p-EMS have not changed in either Finland or Norway since 2014, thus we believe the results still are valid and that newer data would have yielded similar results.

In Norway, 16 physicians participated, and each physician had on average 5 shifts during the data collection period. Each physician registered on average 8 cases, this is on average 1,6 cases per shift. This is a low number if each physician were to be evaluated individually. Because the aim of the study was to evaluate the documentation system, not the individual physician, we find the total number of cases registered in Norway to be acceptable.

The study was conducted in two similar p-EMS settings in two high-income countries and results may not be applicable to all other EMS settings. However, documentation for the study was paper-based, not including expensive equipment. The principles for pre-hospital emergency medical treatment are generally recognized, and international expert consensus on important data to be collected in the field should apply to both low- and high-income EMS systems. The concept of using a template by motivated personnel for data collection may therefore be applicable to other less resource-intensive settings.

Thirteen of the variables are mandatory to register in Finland and electronic patient files cannot be saved unless these variables are registered; completeness rates are therefore 100%. To compare these with Norwegian data will not give an idea of what is possible to collect in an everyday setting or if implementation challenges also apply for this type of data. Finally, the challenge with possible fabricated data to finalize registrations must be addressed.

## Conclusions

We found that a focused data collection method increased data capture compared to a standard data collection method. With a focused data collection method, 88% of variables were more than 80% complete. The greatest deficiencies in completeness rates were evident for physiological parameters. Short missions were associated with lower completeness rates whereas severe illness or injury did not result in reduced data capture. We find the template for p-EMS feasible but highlight motivation and training to maintain high rates of data capture after implementation.

Based on the findings in this study an international consensus-based revision of the template studied will be initiated.

## Additional files

Additional file 1: Template for documenting and reporting in physician-staffed pre-hospital services. A full description of all variables listed in the template for documenting and reporting in physician-staffed pre-hospital services (DOCX 30 kb)

Additional file 2: Definition of severely ill or injured patient. A patient is considered severely ill or injured if one of the listed items are present. (DOCX 17 kb)

## Abbreviations

ASA-PS: American Society of Anaesthesiologists Physical Status Classification
EMS: Emergency Medical Service
FinnHEMS: Finnish Helicopter Emergency Medical Service
IQR: Interquartile Range
MEES: Mainz Emergency Evaluation Score
NAAF: The Norwegian Air Ambulance Foundation
p-EMS: Physician-staffed pre-hospital Emergency Medical Services
SpO2: Saturation of peripheral Oxygen
