# Supplementary material for: Collecting core data in physician-staffed pre-hospital helicopter emergency medical services using a consensus-based template: international multicentre feasibility study in Finland and Norway
Source: BMC Health Serv Res. 2019 Mar 8;19:151. doi: 10.1186/s12913-019-3976-6 (PMC6408770; doi:10.1186/s12913-019-3976-6)
Supplement: Supplementary file 1 — Template for documenting and reporting in physician-staffed pre-hospital services. A full description of all variables listed in the template for documenting and reporting in physician-staffed pre-hospital services (DOCX 30 kb) [file 12913_2019_3976_MOESM1_ESM.docx]

Additional file 1:

Template for documenting and reporting in physician-staffed pre-hospital services

Fixed system variables

| **Data point number** | **Data point name** | **Type of data** | **Data point categories or values** | **Type *** | **Definition of data variable** |
| --- | --- | --- | --- | --- | --- |
| 1 | Speciality of physician | Nominal (categorical data) | 1 = Anaesthesiology  2 = Emergency medicine  3 = Mixed  4 = Other | Bullet list | Mixed refers to both anaesthesiology and emergency medicine |
| 2 | Population | Continuous (numerical data) | Number |  | Annual number of citizens in area covered by service. |
| 3 | Geographical service area provision |  | Squared km |  | Area in which the service is planned to operate, squared km |
| 4 | MD unit hours per year | Continuous | hh:mm |  | Unit hour is defined as the annual sum of hours the unit is occupied on a mission. ∑ time (patient arrival at hospital) - alarm time/year |
| 5 | Mode of transportation accessible for the service | Nominal (categorical data) | 1 = Ground  2 = Rotor-wing  3 = Fixed-wing  4 = Other | Check box | Mode of transportation vehicle(s) available to service on a regular basis. |
| 6 | Operating hours | Ordinal | 1 = Full time service  2 = Day time service  3 = Other | Bullet list | Full time service is operational all days and nights  Part time service is operational only day time and/or in hours of light |
| 7 | Activation criteria | Nominal (categorical data) | 1 = Criteria-based  2 = Consultation with physician  3 = Both | Bullet list | Service is activated in accordance with a pre- defined set of activation criteria used by Emergency Medical Communication Centre  Service is activated only after consultation with an on-call physician |
| 8 | Number of events per year | Continuous (numerical data) | Number |  | Events includes dispatches and requests in which the physician in service is involved when on c |
| 9 | Dispatch system | Nominal | 1 = Integrated Emergency Medical Communication Centre (EMCC)  2 = Special EMCC  3 = Both | Bullet list | Integrated EMCC includes dispatch centres coordinating all levels of pre-hospital services  Special EMCC includes centres only responsible for physician-staffed pre-hospital units |

* “Bullet list” means only one possible answer. “Check box” means multiple answers possible.

hh:mm: hours: minutes

Event operational descriptors

| **Data point number** | **Data point name** | **Type of data** | **Data point categories or values** | **Type** | **Definition of data variable** |
| --- | --- | --- | --- | --- | --- |
| 10a | Call received at Emergency Medical Communication Centre (EMCC) | Continuous | hh:mm |  | The time when the alarm call is answered at the initial EMCC |
| 10b | Unit arrival on scene | Continuous | hh:mm |  | The time when the vehicle stops at a location as close as possible to the patient |
| 10c | Patient leaving scene | Continuous | hh:mm |  | The time when the patient is transferred from the original location or time of death if dead on scene |
| 10d | Patient arrival at hospital | Continuous | hh:mm |  | The time when the patient is formally transferred to receiving medical facility personnel |
| 11 | Type of dispatch | Nominal | 1 = Primary medical mission  2 = Primary trauma mission  3 = Interhospital transfer mission  4 = Search and rescue mission  5= Consultation  6 = Other | Bullet list | Includes all primary missions other than trauma (medical, surgical, paediatric and obstetric) |
| 12 | Type of transportation | Nominal | 1 = Ground ambulance  2 = Helicopter ambulance  3 = Fixed-wing  4 = Other  5 = No transportation | Check box | Main type of vehicle used to transport the patient to definitive care. |
| 13 | Result of dispatch | Nominal | 1 = Patient attended  2 = Mission aborted due to weather  3 = Mission aborted due to technical reasons  4 = Mission aborted not required  5 = Mission aborted alternative tasking  6= Supervision/ advice only | Bullet list | Dispatch means unit alarmed for mission or request/advice/ supervision |

hh:mm: hours: minutes

Patient descriptors

| **Data point number** | **Data point name** | **Type of data** | **Data point categories or values** | **Type** | **Definition of data variable** |
| --- | --- | --- | --- | --- | --- |
| 14 | Age | Continous | Number |  | The patient’s age at the time of event |
| 15 | Gender | Nominal | 1 = Female  2 = Male  3 = Unknown | Bullet list | The patient’s gender |
| 16 | Co-morbidity | Ordinal | 1 = No (ASA-PS = 1)  2 = Yes (ASA-PS = 2-6)  3 = Unknown | Bullet list | ASA-PS definition  1= A normal healthy patient  2 = A patient with mild systemic disease  3 = A patient with severe systemic disease  4 = A patient with severe systemic disease that is a constant threat to life  5 = A moribund patient who is not expected to survive without operation  6 = A declared brain-dead patient whose organs are being removed for donor purposes |
| 17 | Medical problem (main reason for response) | Nominal | 1 = Cardiac arrest  2 = Trauma  3 = Breathing difficulties  4 = Chest pain  5 = Stroke  6 = Acute neurology excluding stroke  7 = Psychiatry including intoxication  8 = Obstetrics and childbirth  9 = Infection  10 = Other | Bullet list | Select the condition most likely to be the patient’s true medical problem |
| 18 | Dominating type of injury | Nominal | 1 = Blunt  2 = Penetrating  3 = Unknown | Bullet list | Indication of the type of injury produced if trauma |
| 19a  b | Glasgow Coma Scale-first  Glasgow Coma Scale- last | Ordinal | Number (3-15)  Number (3-15) |  | First recorded pre-interventional Glasgow Coma Scale upon arrival of physician-staffed service  Glasgow Coma Scale at end of care or patient hand- over |
| 20a  b | Heart rate first  Heart rate last | Continuous | Number (per minute)  Number (per minute) |  | First heart rate per minute measured by physician-  Heart rate per minute at end-of-care or patient hand- over service |
| 21a  b | Systolic blood pressure-first  Systolic blood pressure-last | Continuous | Number (mmHg)  Number (mmHg) |  | First recorded systolic blood pressure measured by physician-staffed service (measured with sphygmomanometer, monitor or intra-arterial line).  Systolic blood pressure at end-of-care or patient handover |
| 22a  b | Cardiac rhythm- first  Cardiac rhythm- last | Ordinal | 1 = Sinus rhythm  2 = SVES, VESmono  3 = Atrial Fib/ Flutt, AV-block gr. II/III, VESpoly  4 = VF, VT, Asystole, PEA  5 = Not recorded  1 = Sinus rhythm  2 = SVES, VESmono  3 = Atrial Fib/ Flutt, AV-block gr. II/III, VESpoly  4 = VF, VT, Asystole, PEA  5 = Not recorded | Bullet list | First cardiac rhythm interpreted by physician-staffed service (minimum 3-channel lead)  Cardiac rhythm at end of care or patient hand-over |
| 23a  b | SpO2-first  SpO2-last | Continuous | Number (0-100)  Number (0-100) |  | First recorded oxygen saturation by physician-staffed service (measured with pulse oxymeter or arterial blood gas (SaO2))  Oxygen saturation at end-of-care or patient hand-over |
| 24a  b | Pain-first  Pain-last | Ordinal | 1 = None  2 = Moderate  3 = Severe  1 = None  2 = Moderate  3 = Severe | Bullet list | First level of pain assessed by physician-staffed service  Level of pain at end of care or patient hand-over |
| 25a  b | Respiratory rate- first  Respiratory rate- last | Continuous | Number (per minute)  Number (per minute) |  | First respiratory rate per minute measured by physician- staffed service. If mechanically ventilated, document ventilation rate.  Respiratory rate at end of care or patient hand-over |

SVES: supraventricular extrasystole, VESmono: single ventricular extrasystole, Atrial Fib/Flutt: atrial fibrillation or flutter, AV-Block: atrioventricular block, VESpoly: polymorphic ventricular extrasystoles, VF: ventricular fibrillation, VT: ventricular tachycardia, PEA:pulseless electrical activity

Process mapping

| **Data point number** | **Data point name** | **Type of data** | **Data point categories or values** | **Type** | **Definition of data variable** |
| --- | --- | --- | --- | --- | --- |
| 26 | Diagnostic procedures | Categorical | 1 = US/Doppler  2 = ECG- analysis (12-lead)  3 = Invasive monitoring  4 = Other point- of-care tests  5 = Point-of-care lab tests | Check box |  |
| 27 | Therapeutic procedures |  |  |  |  |
| 27a | Drugs to facilitate airway procedure | Categorical | 1= Sedatives  2= Neuromuscular blocking agents  3 = Analgesics/ opioids  4 = Local/topic anaesthetics  5 = None | Check box |  |
| 27b | Device used in successful airway management | Categorical | 1=BagMask Ventilation  2 = SAD  3 = Oral TI  4 = Nasal TI  5 = Surgical airway  6 = None  7 = Unknown | Bullet list | Device used for successful airway management (device in place at end of care or patient hand-over) |
| 27c | Breathing- procedures used | Categorical | 1 = Assisted manually  2 = Assisted mechanically  3 = Controlled manually  4 = Controlled mechanically  5 = Chest tube/ decompression  6= Thoracostomy  7 = Other  8 = Unknown | Check box | Open-airway manoeuvres or positioning of patient without use of any technical airway device (chin-lift, jaw thrust, recovery position)  Open-airway manoeuvres, including use of technical devices (guedel pattern, naso-pharyngeal airway)  Breathing assistance using physician’s hands (bag-valve-mask ventilation)  Breathing assistance using technical respiratory support (ventilator, NIV) |
| 27d | Circulation- procedures used | Categorical | 1 = Peripheral IV-line  2 = Central IV- line  3 = IO-Access  4 = Defibrillation  5 =Cardioversion  6 = Pacing  7= Haemostatic, basic  8= Haemostatic, advanced  9 = Other  10 = None | Check box |  |
| 27e | Disability- procedures used | Categorical | 1 = Reduction of fractures  2 = Spinal immobilisation  3 = Therapeutic hypothermia  4 = Other  5 = None | Check box |  |
| 28 | Medication, drugs administered |  | 1 = Yes  2 = No | Bullet list | Indicates whether medication was given by physician-staffed service. Exclude iv-fluid given for “keep-line-open” purposes |
| 29 | Type of medication | Categorical | 1 = Analgesics/ Opioids  2 = Sedatives  3 = Neuromuscular blocking agents  4 = Vasoactive  5 = Fibrinolytic  6 = Antibiotics  7 = Other  8 = None |  |  |

US: ultrasound SAD: supraglottic airway device TI: tracheal intubation NIV: non-invasive ventilation IO-access: intraosseous access

Quality indicators and outcome

| **Data point number** | **Data point name** | **Type of data** | **Data point categories or values** | **Type** | **Definition of data variable** |
| --- | --- | --- | --- | --- | --- |
| 30 | Physiological improvement from SpO2, RR, HR, cardiac rhythm, SBP, GCS and pain |  |  |  | The expert panel recommends developing Quality Indicators based on changes in physiological parameters. |
| 31 | Mission outcome | Nominal | 1 = Left at scene  2 = Patient to hospital, not escorted by physician  3 = Patient to hospital, escorted by physician  4 = Declared dead on arrival at hospital  5 = Declared dead at scene | Check box |  |

SpO2: oxygen saturation RR: respiratory rate per minute HR: heart rate per minute SBP: systolic blood pressure GCS: Glasgow coma scale
