# Supplementary material for: Collecting core data in physician-staffed pre-hospital helicopter emergency medical services using a consensus-based template: international multicentre feasibility study in Finland and Norway
Source: BMC Health Serv Res. 2019 Mar 8;19:151. doi: 10.1186/s12913-019-3976-6 (PMC6408770; doi:10.1186/s12913-019-3976-6)
Supplement: Supplementary file 2 — Definition of severely ill or injured patient. A patient is considered severely ill or injured if one of the listed items are present. (DOCX 17 kb) [file 12913_2019_3976_MOESM2_ESM.docx]

Additional file 2: Definition of severely ill or injured patient

| Deranged physiology  GCS 7 or less  Heart rate 39 per minute or less or 161 per minute or more  Respiratory rate 4 per minute or less or 31 per minute or more  Heart rhythm VT, VF, asystole or pulseless electrical activity  Systolic blood pressure 79 mmHg or less or 230 mmHg or more  Oxygen saturation 85% or less  Advanced medications  Analgesics/Opioids  Sedatives  Neuromuscular blocking agents  Vasoactive  Fibrinolytic  Antibiotics  Advanced procedures  Invasive monitoring  Device used in successful airway management: SAD, Oral TI, Nasal TI or surgical airway  Breathing – procedures used: Ventilation assisted mechanically, controlled manually, controlled mechanically, chest tube/decompression, thoracostomy  Circulation – procedures used: central i.v. line, I.O-access, Defibrillation, Cardioversion, Pacing  Disability – procedures used: reduction of fractures, spinal immobilisation, therapeutic hypothermia |
| --- |
